# Outdoor Fitness Equipment Usage Behaviors in Natural Settings

**DOI:** 10.3390/ijerph16030391

**Published:** 2019-01-30

**Authors:** Hsueh-wen Chow, Dai-Rong Wu

**Affiliations:** Graduate Institute of Physical Education, Health & Leisure Studies, National Cheng Kung University, Tainan 70101, Taiwan; 40105901e@gmail.com

**Keywords:** fitness zone, outdoor gym, sports injury, park, built environment

## Abstract

Outdoor fitness equipment (OFE) areas have become a popular form of built environment infrastructure in public open spaces as a means to improve public health through increased physical activity. However, the benefits of using OFE are not consistent, and several OFE accidents have been reported. In this study, we videotaped how OFE users operate OFE in parks and selected four types of popular OFE (the waist twister, air walker, ski machine, and waist/back massager) for video content analysis. Furthermore, we established coding schemes and compared results with the instructions provided by OFE manufacturers. The results revealed various usage behaviors for the same OFE types. In addition, we observed that a significant portion of user behaviors did not follow manufacturers’ instructions, which might pose potential risks or actually cause injuries. Children are especially prone to act improperly. This study provides empirical evidence indicating the existence of potential safety risks due to inappropriate usage behaviors that might lead to accidents and injuries while using OFE. This study provides crucial information that can be used to evaluate the effectiveness of OFE and to develop future park or open space initiatives.

## 1. Introduction

Although the benefits of physical activity have been well documented in many scientific studies and public health campaigns [1], several surveys conducted by governments worldwide indicated that their citizens do not meet desired physical activity levels [2]. To solve this problem, governments have built physical exercise environments for public use. The effect of public physical exercise environments on citizens’ actual daily physical activity has received considerable attention [3,4].

Among all physical activity–related built environments, parks play a crucial role in addressing the issue of insufficient physical activity because most parks offer open spaces accessible to all, and entry to these parks is free or inexpensive. Parks usually provide easy access within their geographical proximity and are often equipped with exercise facilities, such as swimming pools and ball courts [5]. Recently, many parks worldwide have installed outdoor fitness equipment (OFE) to attract more citizens to visit them and to engage in physical activities [6,7,8,9,10,11,12]. For example, Cohen reported that parks in Los Angeles installed with OFE attracted new visitors and stimulated increased physical activity [6]. In Sydney, the installation of OFE also increased park visits [7]; furthermore, the Health Promotion Service in Sydney partnered with local health districts to promote increased physical activity through the use of OFE [8].

The growing installation of OFE recently can be attributed to the fact that OFE areas have small carbon footprints and are easy to build, practical, and enjoyable [13]. In addition, there is a strong public demand for the provision of active environments that are easily accessible and inexpensive [14,15,16]. The installation of OFE in parks or open spaces has offered many benefits, including attracting new visitors and increasing the overall number of park visits [6,7,8], increasing engagement in physical activities [12,15], improving perceptions of security [12], adding pleasant contributions to cities’ landscapes [12,17,18], and encouraging social interaction [7,15].

Only in the past decade have studies related to OFE directly addressed the benefits of using OFE to individuals. These benefits include physical benefits, such as improved cardiorespiratory fitness, muscle strength, balance, and flexibility [8,19,20]. However, to date, these studies have reported limited and mixed results. For example, Nguyen and Raney found that after engaging in OFE training, participants’ weight, body fat percentage, and waist circumference decreased significantly and muscular endurance repetitions increased significantly. However, they did not observe an increase in the index of cardiovascular ability (VO_2_ max). This finding might be attributed to the fact that unlike machines in indoor gyms, the resistance levels of most OFE cannot be adjusted to increase the training effect [11]. Chow studied the effect of the intervention of OFE on older adults and reported significant improvements in chair standing and 8-foot up-and-go tests when baseline (pretest) findings were compared with post-test findings; however, these improvements decreased significantly from the post-test to the retention test. No significant changes were detected for arm curl, 2-min walk, chair sit-and-reach, and back scratch tests [21].

The benefits of OFE have not yet been fully explored, and adequate scientific results are not available to support the idea that OFE can provide benefits such as increased aerobic and muscular strength or balance and flexibility. The reason for this might be due to the presence of insufficient specific instructions for using OFE, because a study that inspected all OFE facilities in Taipei and Tainan parks found inadequate instructional labels for the proper use of equipment and recommended durations or repetitions [22]. Moreover, an observational study indicated that many users use OFE only for very short periods of time, which are insufficient to produce substantial health benefits [23]. 

Several studies have indicated that OFE poses many safety problems because of a lack of surveillance and inadequate usage instructions. For example, a study reported that 79% of OFE users developed health problems after using OFE and that 54% complained of muscle pain [24]. In addition, several studies have reported that many OFE accidents or sports injuries occurred because users operated equipment incorrectly [25,26]. Xie found that 66.5% of OFE users in China reported being injured after using OFE. Major factors attributing to injuries were the malfunction of equipment (58.3%) and improper usage behavior (31.4%) [25]. While examining accidents or injuries related to parks, many studies conducted between 1970 and 1990 have considered only playgrounds for children. Many severe accidents, resulting in fractures, concussions, head injuries, use of ambulance services, and extended hospital stays, have been reported. Studies have identified factors related to playground injuries, including the playground environment, behaviors, and usage frequency, and such studies have reported that after guidelines were established for playground safety (i.e., playground surface and height), injuries associated with playgrounds decreased considerably [27,28,29,30].

Because the installation of OFE is a new feature in parks or open spaces in many regions worldwide, a limited number of studies have investigated the risks of these types of equipment based on operating methods and patterns. Observing how people use OFE in real settings can assist in identifying problems that cause OFE accidents and injuries. Therefore, the present study was conducted to understand OFE usage behaviors. The specific aims of this study were (1) to observe OFE usage behaviors in real settings and to classify these behaviors, (2) to determine differences between OFE usage behaviors and instructional behaviors, (3) to investigate whether differences in gender and age groups can lead to different behaviors when using OFE, and (4) to assess the potential risks of each OFE usage behavior.

## 2. Materials and Methods

In this study, we performed video content analysis, where video recordings of OFE users’ behaviors were examined and classified into different movement patterns. 

### 2.1. Video Content Analysis

To determine OFE usage behaviors in a natural environment, we used a video recording method to track users’ explicit behaviors. These subjects had come to use the OFE on their own volition. These videos allowed us to review actual OFE use and to conduct a scientific analysis of events of interest [31]. Video data analyzed in this study were obtained from an earlier study, in which park visitors in two parks in Tainan City, Taiwan, were videotaped while using OFE [22]. In brief, these videos were filmed in Xihu Park in the summer of 2012 and in Dongning Park in the summer of 2015. Data were collected during 2-h peak periods in the morning and afternoon to observe more users. Videos were recorded on both weekdays and weekends to include a wide range of user backgrounds because more children and youths visited parks during the weekend. Additional details were described elsewhere [23]. A total of 28 h of video footage was analyzed in this study. 

These videos were valuable in identifying the patterns of OFE use because these recordings recorded individuals’ real behaviors in a field environment instead of in a controlled laboratory setting. Recordings used in this study were approved by the Internal Review Board of National Cheng Kung University (IRB_ER-99-375) [23], and the analysis of materials was approved by the Human Ethics Committee of National Cheng Kung University (REC_105-065). The Mangold INTERACT software program (Interact; Mangold International GmbH, Arnstorf, Germany), a professional behavioral analysis tool, was used in this study because of its three advantages: (1) objectivity (the analysis is objective); (2) repeatability (replicable results can be obtained from the same conditions); and (3) comprehensibility (findings are easily understandable). This software is a multifunctional platform that can implement behavioral event coding, behavioral data graphing, and analysis [31]. 

### 2.2. OFE Equipment for Analysis

In this study, a waist twister (Colisy; Community Lifestyle Co. Ltd., Taipei, Taiwan), air walker (HUNGJWU Co., Ltd., Tainan, Taiwan), ski machine (Colisy; Community Lifestyle Co. Ltd., Taipei, Taiwan), and waist/back massager (Timix; Community Lifestyle Co. Ltd., Taipei, Taiwan) were selected as our targets for analysis, because these four types of equipment were popular among users [22,23]. Each OFE user who performed movements on these four equipment types was coded. Each individual was identified by gender (male/female), age group (children/youth/adults/seniors), and usage behaviors and durations. In addition, because individuals might change their OFE usage behaviors for the same equipment, the total behavior counts were recorded.

### 2.3. Coding Methods

To classify usage behaviors, we first checked the labels or operation instructions provided by manufacturers for each OFE. If the behavior followed the instructions, users were coded as having followed the indicated behavior. For example, the designed motion of the triple waist twister is to “turn the waist,” so the indicated behavior of the triple waist twister is “standing in front of equipment to turn the waist” and was coded as code WT-1. Behaviors that were nonindicative were coded based on how individuals used each piece of OFE. A description for each behavior code was created to illustrate each behavior (see Table A1, Table A2, Table A3 and Table A4). 

To ensure the reliability of categorizing usage behaviors among different researchers, an inter-rater reliability test was performed between independent raters to assess the consistency with which they rated users’ gender, age, and behaviors while using OFE. All raters had received professional training in the use of the Mangold INTERACT software program (Mangold International GmbH, Arnstorf, Germany). In this study, the main rater first reviewed all videos, created coding schemes, and shared the schemes with the second rater. Then, an hour-long video recording for each type of equipment was randomly selected to examine inter-rater reliability. The resulting Cohen’s kappa value acquired within the software averaged 0.83, 0.75, and 0.84 for gender, age, and behavior, respectively. These results were considered to have acceptable reliability. 

### 2.4. Data Analysis

Statistical analysis was performed using SPSS (IBM statistics v.22.0, Armonk, NY: IBM Corp.) for descriptive statistics and crosstab Pearson’s chi-square test to compare proportions of the (non)indicative OFE behaviors of different gender and age groups. Significance levels were set at 0.05. The Bonferroni method was used in post-hoc tests, with correction for alpha inflation, for comparing distributions among four age groups. Furthermore, R software was adopted to conduct Fisher’s exact test when the expected frequency was less than five in any group. 

### 2.5. Expert Panel Interview

An interdisciplinary panel of experts from the backgrounds of physical therapy, sports science, fitness training, and mechanical engineering was invited to critically evaluate potential sports injuries or accidental risks and benefits of each usage behavior. The videos of each OFE behavior coded in the early phase of this study were presented to the experts during the interview session, and discussions were tape-recorded and transcribed for data analysis. General themes for overall OFE use and for each identified behavior that emerged from the discussion were recognized. To ensure the accuracy of data and analysis, all transcribed data and a summary of expert panel interviews were returned to each expert for confirmation.

## 3. Results

### 3.1. User Characteristics

**Figure 1:**
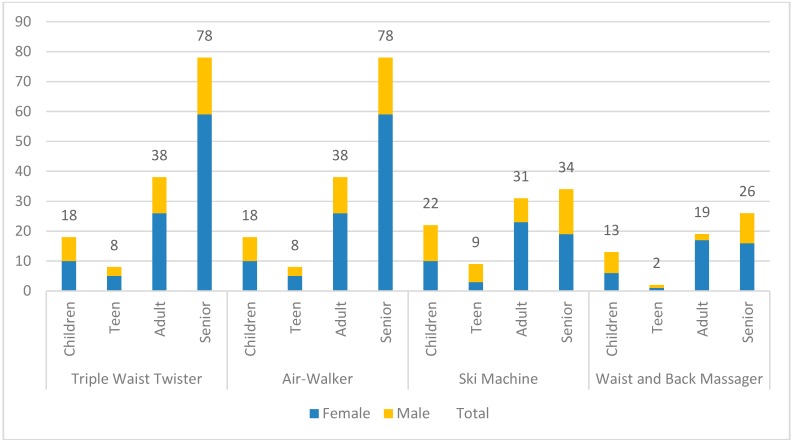
Observed outdoor fitness equipment (OFE) user counts in terms of age and gender for each piece of equipment.

Among the four types of OFE analyzed in this study from video recordings, the air walker (n = 195) had the highest user count, followed by the triple waist twister (n = 142), ski machine (n = 96), and waist/back massager (n = 60). More female OFE users (63%) than male OFE users were observed. In terms of age group, seniors represented the highest proportion of users (47%). The distribution of gender and age in terms of the four types of OFE is shown in Figure 1.

### 3.2. User Behaviors of OFE

**Figure 2:**
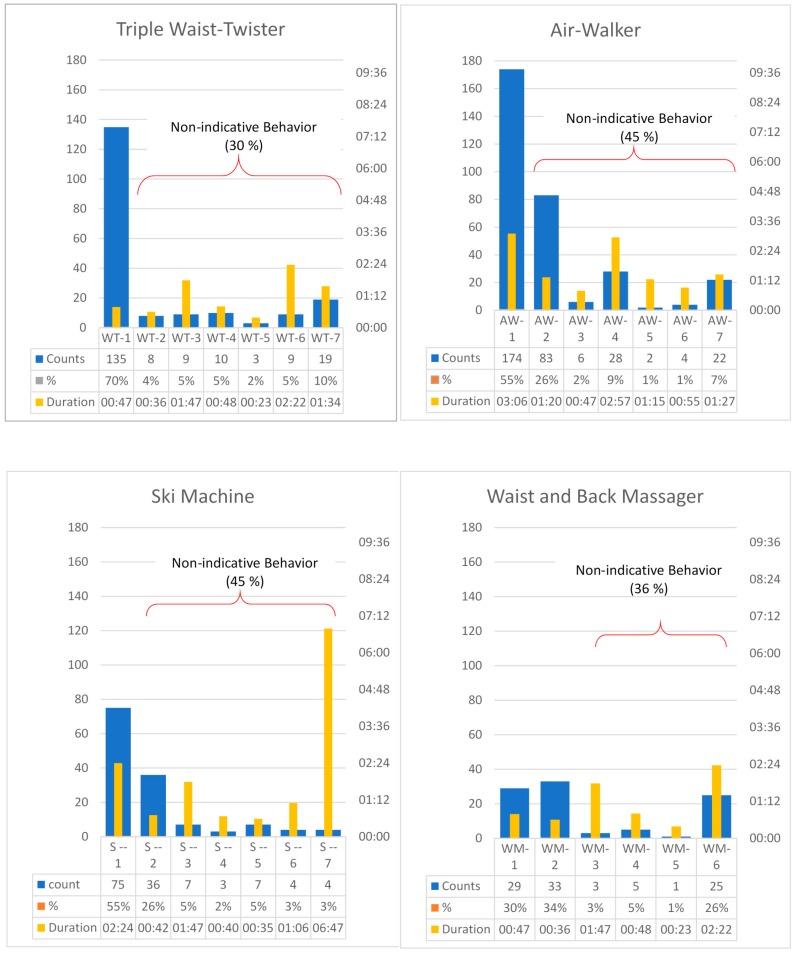
Coding schemes and observed counts, percentages, and duration for four OFE.

From the field setting, we observed that OFE users used the same equipment in different manners. For example, for the air walker, most users strode in the reverse direction, standing in front of the equipment (AW-1), whereas other users used two legs to stride in the same direction simultaneously (AW-2), and others exercised with their back turned to the equipment (AW-3). We categorized seven types of user behaviors for the triple waist twister, air walker, and ski machine, and six types of user behaviors for the waist/back massager (see Appendix for detailed descriptions of movement patterns for each coding scheme). The observed frequencies for each type of behavior are presented in Figure 2. In addition, individuals might change their behavior for the same equipment. For example, one might use the air walker in AW-1 behavior and later change to AW-2 behavior; this was recorded as once for the user count (same individual) but twice for the behavior count (1 in AW-1 and 1 in AW-2). The same person using multiple types of equipment was coded for one user count but was coded for multiple behavior counts for behaviors identified for each observed OFE. Therefore, we also summarized behavior counts for each piece of equipment (please see Figure 2 & Table 1). All behavior counts were higher than user counts, indicating that many users changed their behaviors for a single type of equipment. The average durations of most behaviors were very short. 

### 3.3. Percentage of Indicative OFE User Behaviors

We further classified behaviors as “indicative behaviors” if they were in line with instructions provided by equipment manufacturers, whereas all other behaviors were classified as “nonindicative behaviors.” User behaviors for the triple waist twister (WT-1), air walker (AW-1), and ski machine (S-1) that complied with manufacturers’ instructions were coded as 1, and user behaviors for the waist/back massager (WM-1 and WM-2) that complied with manufacturer’s instructions were coded as 1 or 2. Nonindicative behaviors accounted for 30% of our observations for the triple waist twister, 45% for the air walker, 45% for the ski machine, and 36% for the waist/back massager (Figure 2).

### 3.4. Differences in OFE Usage Behaviors among Different Genders and Age Groups

**Figure 3:**
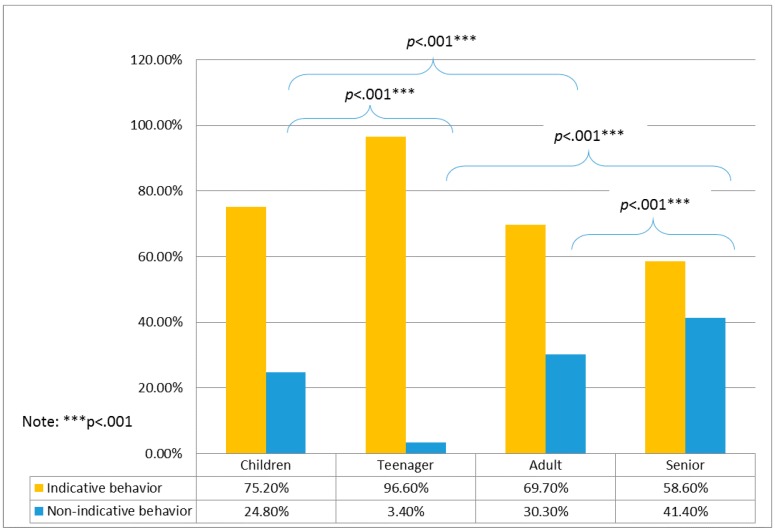
Post-hoc test results of user groups and indicative behaviors for the air walker.

**Figure 4:**
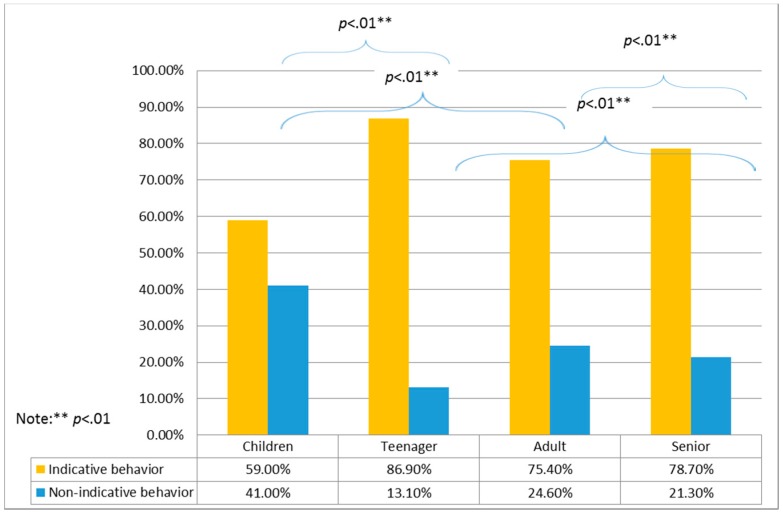
Post-hoc test results of user groups and indicative behaviors for the ski machine.

The results of the chi-square test showed no relationship between gender and the frequency of nonindicative behaviors (Χ^2^_triple waist twister_ = 1.506, *p* = .22; Χ^2^_air walker_ = 0.113, *p* = .736; Χ^2^_ski machine_ = 2.4, *p* = .121; Χ^2^_waist and back massager_ = 0.016, *p* = .9). For age groups, significant differences were found only for the air walker and ski machine (Table 1). For both the air walker and ski machine, a higher percentage of nonindicative behaviors was observed in children and seniors than in those in other age groups (Figure 3 and Figure 4). 

### 3.5. Results of Expert Panel’s Evaluations

From the discussion and evaluation of the expert panel, four major themes for proper OFE use were identified. These include (1) design and safety, (2) correct posture and operation, (3) individual’s ability, and (4) alternative behaviors. First, the experts found that many pieces of OFE in Taiwan are equipped with unsteady pedals or platforms and do not have adjustable resistance functions. Therefore, OFE users require careful control of their center of gravity to maintain balance and prevent a fall. Users who use equipment with no resistance function tend to stretch over their limit or perform movements rapidly without stopping. Thus, extra caution should be taken. Second, the expert panel believed that although the design of OFE is not ideal, if users are mindful of operating the equipment with correct postures and operation methods, they can achieve desired training goals. By contrast, if users do not use the equipment correctly, for example, if users always stretch their joint too far, exceeding their maximal degree of motion, it is likely to result in injuries in the long term. Third, the expert panel emphasized that major differences exist in terms of fitness levels or skills among users. Thus, individuals should adjust intensities or durations based on their abilities. Finally, after reviewing all coded behaviors, the experts agreed that not all nonindicative behaviors are risky. For example, WM-6, wherein users performed upper body pushups on the waist and back massager, is considered harmless and accepted by experts as the training goal is to increase upper body strength instead of massage. The expert panel recommended that it is essential for users to first recognize the training goals (i.e., endurance, muscular strength, flexibility, or balance) of each behavior, then select proper equipment, and finally perform the behavior with correct postures and operation methods. Misuse of OFE might eventually result in injuries that offset the good intention of increasing physical activity for the benefit of public health. The expert panel also concluded that safety should be the first priority for related authorities. 

## 4. Discussion

### 4.1. Various Types of OFE Usage Behavior

The provision of OFE has become a popular built environment infrastructure choice in public open spaces as a means to improve public health through increased physical activity [10,14,32]. To our knowledge, no study has investigated the actual use of OFE, even though this can provide crucial information for evaluating the effectiveness of OFE and develop future park or open space initiatives. The results of this study revealed that participants presented a variety of behaviors for the same OFE. For example, for the air walker, seven different behaviors were identified based on our real field video recordings and coding schemes, indicating that many users did not follow indicative behaviors labeled by manufactures. A previous interview study indicated that many users merely mimic how others use the equipment because no information session was conducted after installing the OFE and many instructions were absent [15]. In addition, we found that some users may change their behaviors while using the same OFE. This might be because users felt sore after using the same posture/pattern, users wanted to increase the difficulty of using the OFE, or users wanted to explore different behaviors for fun. The design of the equipment cannot restrict users to particular approaches to using OFE, which can lead to potential injuries due to several risky nonindicative user behaviors. The results are also in line with previous research indicating that users operated the OFE only for short periods of duration [23]. 

### 4.2. Potential Risks of OFE Usage Behaviors

Among various OFE usage behaviors, although some nonindicative behaviors were found to be harmless, many behaviors presented a potential safety risk. The results of this study revealed that user behaviors for the triple waist twister, air walker, ski machine, and waist/back massager respectively followed manufacturers’ instructions only 70%, 55%, 55%, and 64% of the time that they were used. This finding indicates that approximately half of the user behavior counts for the air walker and ski machine did not follow suggested instructions for preventing risks or injuries. The expert panel identified that many users could lose their balance because they stood on only one pedal (e.g., AW-6 and S-5) or they did not face the equipment (e.g., WT-2, AW-3, AW-5, AW-6, S-5, and S-6). Some users strode too fast or the amplitude of their sway was too large (e.g., AW-2, AW-4, AW-5, S-2, and S-4); these actions may strain or sprain the lower body or waist because the sudden stretching of ligaments above users’ limits is likely to tear muscle fibers or tendons. Some users twisted their waists too fast or exceeded the twisting angle appropriate for that individual (e.g., WT-5 and WT-6); these actions may strain or sprain the waist because it is pulled too far out of its normal range. 

Using OFE correctly is important to prevent injuries or other health problems. We did not observe any confirmed injuries from our video data. However, several news reports have described OFE accidents [33,34]. Attention should be focused on improper OFE usage behavior because it might not result in acute sports injuries or severe accidents immediately, but it can more likely result in chronic sports injuries or pain over time. Studies have indicated that many OFE sites do not have instructional signage or users do not pay attention to instructions that are provided, which can lead to accidents resulting from the misuse of OFE [15,23]. Because of misuse or inappropriate actions, the health benefits associated with the installation of OFE may not be achieved. In addition, several chronic or acute injuries resulting from misuse of OFE can limit or prevent physical activity participation, leading to poor health.

### 4.3. OFE Usage Behaviors: Age Differences

In this study, most of the users were seniors (47%); this finding is in line with those of previous studies [8,20,23]. In addition, a high proportion of seniors performed nonindicative behaviors. This might be because many older adults lack the knowledge or confidence to use OFE correctly [15]. In addition, our results showed that many children were interested in using OFE, even though many OFE sites or fitness zones are designed for adults. Compared with other age groups, children are more likely to be attracted to play equipment and tend to be more physically active in the open spaces of a public park [35,36]. The findings of this study revealed that children were more likely to perform nonindicative behaviors than other age groups. Therefore, children are more prone to be injured. Many pieces of OFE are not designed for children, and numerous fitness zones equipped with OFE have set age restrictions. However, because many of these pieces of OFE are placed in non-supervised open spaces, such as parks or open green spaces, it is difficult to prevent children from using OFE even if it has clear instructions indicating the minimum age and height requirements for users. Such equipment is usually not suitable for children and may jeopardize their safety. 

OFE has been popular as a means for promoting public health by engaging the public in active living. To our knowledge, this is the first study to provide empirical data regarding OFE usage behaviors. This study has identified, in real settings, user behaviors in various forms while using four popular types of OFE. The study results indicated that a significant number of behaviors in the field do not follow instructions provided by manufacturers for operating their OFE. Although some alternative behaviors are anodyne, most of the observed nonindicative behaviors were not safe, as indicated by the experts. This study has raised crucial questions regarding the safety of using OFE, which have significant implications for governmental policies and manufacturers. Because several OFE accidents have been reported [33], it is important for the government and for manufacturers to carefully consider these safety issues while installing this type of equipment in public places.

## 5. Conclusions

The installation of OFE in public spaces has become increasingly popular as a means to encourage the public to become more physically active and socially connected. However, this study provides empirical evidence indicating that there are potential safety risks in the manner in which many people use OFE, which might lead to accidents and injuries that can result in issues of liability for manufacturers or related authorities. The results of this study suggest that manufacturers should provide clear equipment operation guides (or demonstration videos) on the correct use of their equipment and warning messages regarding risky behaviors. Manufacturers should also design OFE with suitable ranges of swing angles or fixed operating positions. Governments or local authorities that authorize or sponsor the installation of OFE might conduct instructional sessions in which professional trainers can explain how to use OFE properly, safely, and effectively in order to meet each individual’s capability and fitness level [8,10,16]. This is especially required for older adults who might lack confidence when using the equipment [9,37]. Information sessions should also target parents with children, emphasizing the risks posed to children in adult-only OFE areas. These instructional sessions can also serve as marketing strategies to attract park visitors or to promote new OFE sites [8]. 

Two limitations of this study should be noted. First, because we used video recordings in real settings instead of in a controlled laboratory, coding schemes could not objectively measure the details of behaviors in terms of the twisting angle, the amplitude of sway, the range of motions of a joint, or operating speed. Second, we analyzed only the manner in which people used the four popular OFE types in Taiwan. Thus, our results may not be fully generalizable to other equipment types or regions. Despite these limitations, the findings of this study have many crucial implications for future practice. For example, governments can formulate clear policies and regulations concerning safety issues before building fitness zones or installing OFE. Manufacturers can design and develop equipment that meets certain principles of safety, ergonomics, and maintainability. Park authorities can conduct sessions to provide user guidelines to the public in the community and implement effective promotional strategies. For example, governments can attach a label to the equipment with a quick response (QR) code that links to a video demonstrating how to use each OFE appropriately. Park managers can post warning signs to restrict children from using OFE and routinely monitor and manage OFE areas.

This study has raised many questions that require further investigation. Future research should explore other types of OFE and other user behaviors and determine whether different OFE designs lead to different patterns of use. For instance, studies can determine whether an OFE with a swing function (e.g., an air walker) can more likely result in nonindicative behaviors. Furthermore, future research should broadly survey OFE users or those who quit using OFE in terms of their OFE-related injury/accident experience and identify behaviors that are associated with these accidents. Another study could invite participants to perform different OFE behaviors observed in this study and use electromyography to detect the activation of corresponding muscles to clearly illustrate the effect of different behaviors on the human body and identify potential risks or benefits and the physical effectiveness inherent in each of these fitness training behaviors. This research has identified several behaviors commonly used by the public while using OFE and has offered many opportunities for future scientific inquiry. Ensuring the safety of OFE users should be a priority when promoting public health through the encouragement of active lifestyles.

## Figures and Tables

**Table 1:** Pearson’s chi-square test for the age group and non (indicative) behavior for each type of equipment.

OFE		Triple Waist Twister		Air Walker		Ski Machine		Waist/Back Massager	
Users counts		142		195		96		60	
	Behaviors	Indicative behavior Count (%)	Non- indicative behavior Count (%)	Indicative behavior Count (%)	Non- indicative behavior Count (%)	Indicative behavior Count (%)	Non- indicative behavior Count (%)	Indicative behavior Count (%)	Non- indicative behavior Count (%)
Age groups									
Children		15 (7.8%)	7 (3.7%)	10 (3.2%)	36 (11.3%)	11 (8.0%)	25 (18.4%)	8 (8.3%)	9 (9.4%)
Teenager		7 (3.6%)	2 (1.0%)	6 (1.9%)	5 (1.5%)	7 (5.1%)	8 (5.9%)	2 (2.0%)	0 (0%)
Adult		38 (19.7%)	20 (10.3%)	65 (20.4%)	44 (13.8%)	26 (19.1%)	15 (11.0%)	23 (24.0%)	12 (12.5%)
Senior		75 (38.9%)	29 (15.0%)	93 (29.2%)	60 (18.8%)	31 (22.8%)	13 (9.6%)	29 (30.3%)	13 (13.5%)
Total (=Behavior counts)		135 (70%)	58 (30%)	174 (54.7%)	145 (45.4%)	75 (55%)	61 (44.9%)	62 (64.6%)	34 (35.4%)
Chi-squared		1.07		23.51 ***		14.54 **		3.76	
*p*-value		.8052		.0005		.002		.363	

OFE—Outdoor fitness equipment, Note: ** *p* < .01, *** *p* < .001.

## Appendix A

**Table A1:** Depictions of user behaviors for the triple waist twister.

Coding Number	Behavior	Movement
WT-1	Turn the waist, standing in front of equipment	
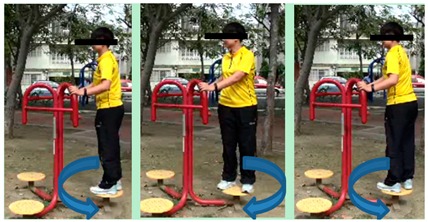
WT-2	Turn the waist, standing with back turned to the equipment	
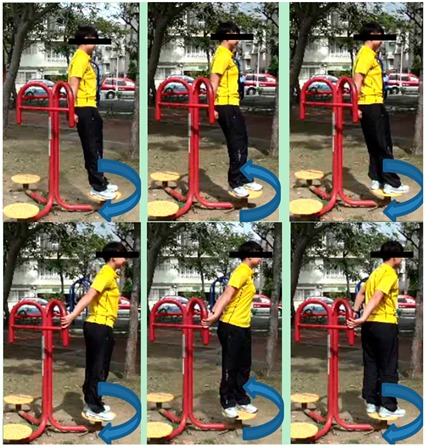
WT-3	Turn the waist, squatting in front of the equipment	
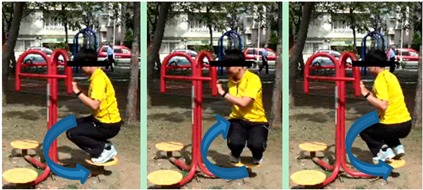
WT-4	Lower body exercise	
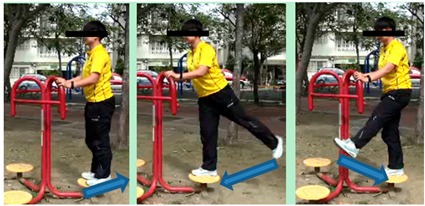
WT-5	Rotation: 360 degrees	
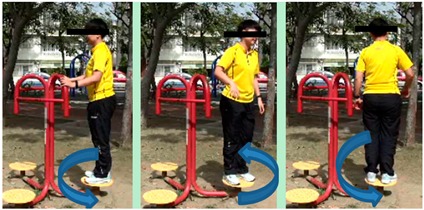
WT-6	Both hands sway while rotating 360 degrees slowly or rotating randomly in any degree range	
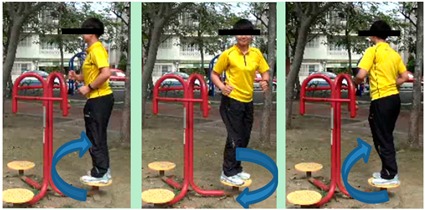

**Table A2:** Depictions of user behaviors for the air walker.

Coding Number	Behavior	Movement
AW-1	Stride in reverse direction, standing in front of equipment	
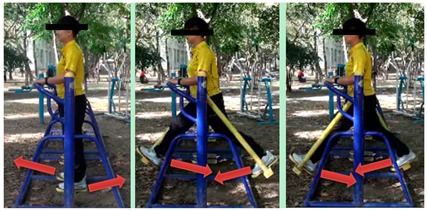
AW-2	Two legs stride in the same direction	
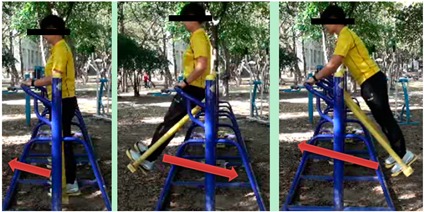
AW-3	Stride in reverse direction with back turned to the equipment	
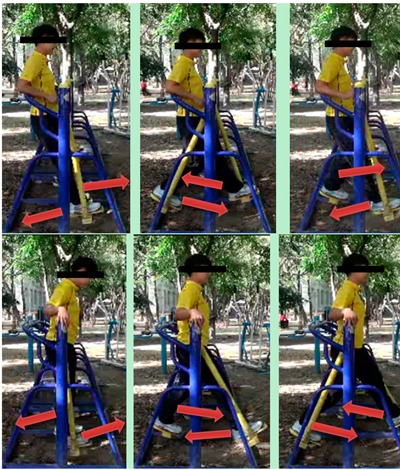
AW-4	Stride with one leg, standing in front of the equipment	
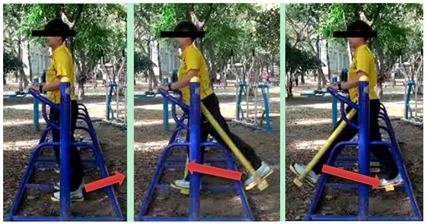
AW-5	Stride with one leg with back turned to the equipment, another leg standing on the land	
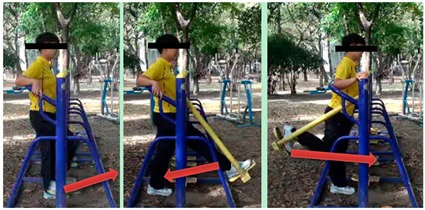
AW-6	Stand on the same pedal with side lateral to stride	
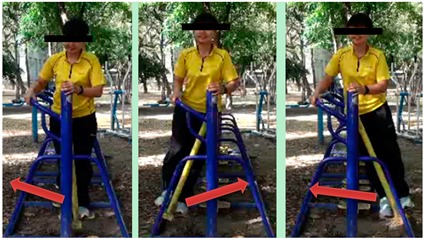

**Table A3:** Depictions of user behaviors for the ski machine.

Coding Number	Behavior	Movement
S-1	Stride side-to-side with using hands and legs	
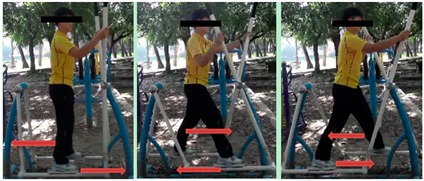
S-2	Stride with hands and legs using the same side	
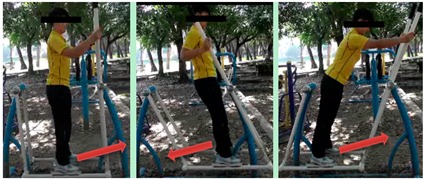
S-3	Stride with legs side-to-side, hands are doing other things or not holding the grips	
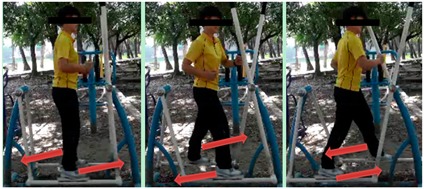
S-4	Stride with one hand and one leg side-to-side	
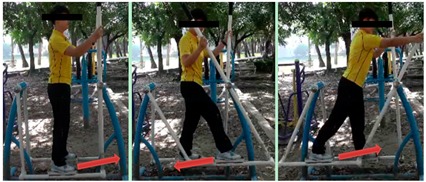
S-5	Stand on the same pedal with lateral side to stride movement	
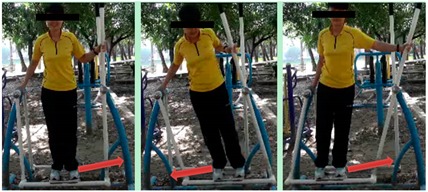
S-6	Stride with side-to-side movement with hands and legs and back turned to the equipment	
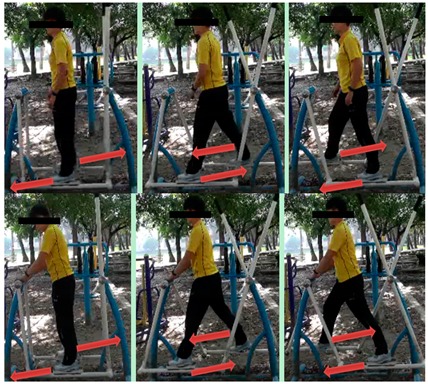

**Table A4:** Depictions of user behaviors for the waist/back massager.

Coding Number	Behavior	Movement
WM-1	Massage back or waist in the vertical direction	
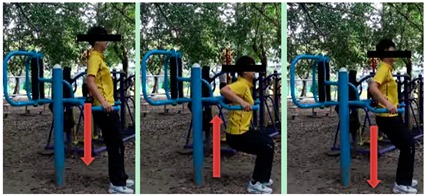
WM-2	Massage back or waist in the horizontal direction	
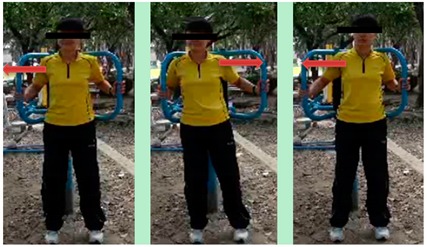
WM-3	Massage waist with upper body leaning forward and laying back	
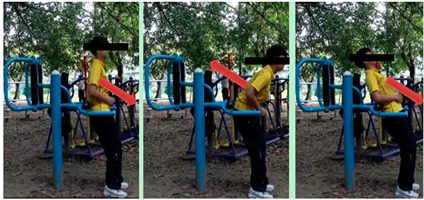
WM-4	Massaging hands	
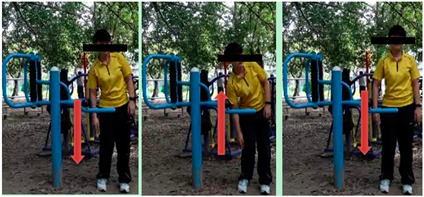
WM-5	Massaging abdomen	
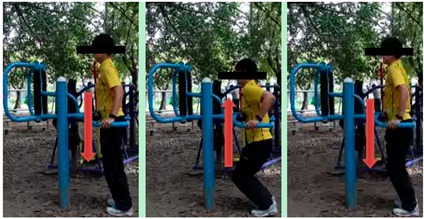
WM-6	Others (Upper body stretching exercise; Upper body exercise; Chatting or taking a break in any posture)	(Upper body exercise)
